# Bibliography

**Published:** 1892-09

**Authors:** 


### Bibliography.

Annual of the Universal Medical Sciences. Edited by Charles E. Sajous, M.D., and seventy associate editors, assisted by over two hundred corresponding editors, etc. Philadelphia, New York, Chicago, and London: F. A. Davis Company, 1892.

With a promptness that does credit to the editor, editorial staff, and publisher, this Annual, in five volumes, is given to the medical reading world. The value of these for reference has ceased to be a question. They have become so important that the wonder grows steadily each year why this compilation of facts was not begun years ago.

The editor truly says in his preface, "It does more than any publication extant for the perpetuation of the journals and of the work presented in them. It carries their names and the good work they contain to all parts of the earth, and, between covers calculated to ornament a library, preserves their identity and their choice contributions for future generations. How many complete series of that once great journal, *London Times and Gazette*, could now be found in the libraries of medical men? Had the Annual then lived most of its talented contributors, now dead to contemporary medicine, would still live through their work; whereas now the difficulty of obtaining access to their writings relegates them to the domains of obscurity."

It is strange, in view of these facts, that a persistent opposition exists to this publication on the part of several medical periodicals.

It is beyond the power of any single individual to read the medical journals now published in all languages, and, therefore, no greater service can be rendered than the condensation of facts from all sources, as presented in these volumes. The work is so well done by the large staff that the yearly advent is looked forward to as furnishing the most valuable series of reference-books published during the year.

Considerable space is given to "Oral and Facial Surgery," by Rudolph Mates, M.D. Full reports of cases, with illustrations, are presented, taken from the dental and medical journals, and furnish a chapter of great value to those interested in this specialty.

While the editor states "no improvements are announced this year," it seems to us that the publication has reached a point of stability where few changes are needed, and we, therefore, hope it may be continued practically as it is,—one of the most useful contributions to medical literature, and indispensable to every well-selected medical library.

